# Urokinase-type plasminogen activator receptor (uPAR) on tumor-associated macrophages is a marker of poor prognosis in colorectal cancer

**DOI:** 10.1002/cam4.242

**Published:** 2014-05-30

**Authors:** Martin Illemann, Ole Didrik Laerum, Jane Preuss Hasselby, Tine Thurison, Gunilla Høyer-Hansen, Hans Jørgen Nielsen, Ib Jarle Christensen

**Affiliations:** 1 The Finsen Laboratory, Rigshospitalet, Copenhagen University HospitalCopenhagen, Denmark; 2 Biotech Research and Innovation Center (BRIC), University of CopenhagenCopenhagen, Denmark; 3 Department of Pathology, Rigshospitalet, Copenhagen University HospitalCopenhagen, Denmark; 4 Department of Surgical Gastroenterology, Hvidovre HospitalHvidovre, Denmark

**Keywords:** Colorectal cancer, immunohistochemistry, invasion, survival, uPAR

## Abstract

Patients were identified from a population-based prospective study of 4990 individuals with symptoms associated with colorectal cancer (CRC). A total of 244 CRC tissue samples were available for immunohistochemical staining of uPAR, semiquantitatively scored at the invasive front, and in the tumor core on cancer cells, macrophages, and myofibroblasts. In addition, the levels of the intact and cleaved uPAR-forms in blood from the same patients are evaluated in this study. In a univariate analysis, the number of uPAR-positive versus uPAR-negative macrophages (HR = 2.26, [95% CI: 1.39–3.66, *P* = 0.0009]) and cancer cells (HR=1.49, [95% CI: 1.01–2.20, *P* = 0.047]) located in the tumor core were significantly associated to overall survival. In a multivariate analysis, uPAR-positive versus uPAR-negative macrophages located in the tumor core showed the best separation of patients with positive score associated to poor prognosis (HR = 1.84 [95% CI: 1.12–3.04, *P* = 0.017]). In a multivariate analysis including clinical covariates and soluble uPAR(I), the latter was significantly associated to overall survival (HR = 2.68 [95% CI: 1.90–3.79, *P* < 0.0001]) and uPAR-positive macrophages in the tumor core remained significantly associated to overall survival (HR = 1.81 [95% CI: 1.08–3.01, *P* = 0.023]). Membrane-bound uPAR showed additive effects with the circulating uPAR(I) and stage, giving a hazard ratio of 12 between low and high scores. Thus, combining stage, uPAR(I) in blood and uPAR on macrophages in the tumor core increase the prognostic precision more than tenfold, as compared to stage alone.

## Introduction

Colorectal adenocarcinomas constitute a complex environment of stromal elements apart from a rather heterogeneous tumor cell population (for review, see 1). This implies that in some cases the presumably normal “appearing” accessory cells may outnumber the malignant cells. Although immune reactions against the malignant cells are common, the immune cells—and mainly macrophages—may become polarized to collaborate with the neoplastic cell population and thus contribute to the malignant behavior 2. In addition, the formation of local blood vessels as well as connective tissue is necessary for tumor invasion, progression, and dissemination 1.

Recently, we have examined the importance of the plasminogen-activating system in adenocarcinomas in other locations of the gastrointestinal tract (GI-tract). Activation of plasminogen to plasmin in the tumor microenvironment leads to a cascade of proteolytic activities in addition to malignant cell migration and angiogenesis, thus enhancing invasion and dissemination 3. The urokinase-type plasminogen receptor (uPAR) is of particular importance for this process, as receptor binding is a prerequisite for pericellular plasmin formation, which is required for tissue remodeling during cancer invasion 4. Increased expression of uPAR is most likely associated to increased invasive capability in different malignant tumors. The expression and localization of uPAR in tumor tissue may thus be of clinical importance 5.

In gastric adenocarcinomas, we found that uPAR-expression on a high percentage of the malignant cells at the invasion front of the tumor was associated with poor prognosis 6. In adenocarcinomas of the lower esophagus, a cancer type with a dismal prognosis a high proportion of uPAR-positive cells was found among cancer cells, macrophages, and myofibroblasts. uPAR-upregulation was even seen in nerve bundles close to the tumor. This indicates that there is a strong local stimulus for uPAR-expression in the microenvironment of the invasive area. In this type of cancer, the number of uPAR-positive malignant cells in tumor core and the number of uPAR-positive macrophages at the invasive zone were associated to a worse prognosis. In the invasion zone, the cancer tissue showed deep penetration into the esophageal wall and surrounding tissue 7.

In colorectal cancer (CRC), uPAR has been localized to the invasive front and expressed mainly by macrophages but also expressed by some myofibroblasts and by a few cancer cells, the so-called budding cancer cells 8,9. The prognostic significance of uPAR-forms in tumor-tissue from CRC patients has previously been determined by immunoassay quantification in tumor-tissue extracts 10. The protein consists of three domains and is attached to the cell membrane via a glycolipid anchor. On the cell surface, intact uPAR [uPAR(I–III)] is cleaved, liberating the amino-terminal domain I [uPAR(I)], and leaving the cleaved uPAR(II-III) on the cell surface. The two cell surface-bound forms can be shed and thus three soluble forms of uPAR can be identified in the blood 11. The functions, if any, of soluble uPAR(I–III) and uPAR(I) are not clarified, whereas soluble-cleaved uPAR(II–III) have been demonstrated to be involved in chemotaxis 11. The combined amount of the soluble uPAR-forms in plasma from CRC patients is a strong prognostic marker with high levels correlating to poor prognosis 12, and interestingly the liberated uPAR(I) and the uPAR(I–III) + uPAR(II–III) are independent prognostic markers 13. The levels of the cleaved uPAR-forms could reflect pericellular proteolytic activity from the tumor, and thus be a measure of invasive activity.

We here present evidence that although uPAR is present on both cancer cells and myofibroblasts, expression on macrophages seems to be a substantial prognostic factor. Furthermore, when the number of uPAR-positive macrophages in tumor core was combined with the plasma level of uPAR(I) and stage, this separated the patients into distinct groups with several fold differences in overall survival.

## Materials and Methods

### Patients

From November 2003 through December 2005, patients were included in a multicenter cross-sectional study conducted at six Danish hospitals. Eligible for inclusion were patients (aged 18+ years) undergoing endoscopic examination following symptoms related to CRC and patients attending surveillance programs due to hereditary CRC 14. A total of 303 patients were diagnosed with CRC. Routine-fixated paraffin-embedded tumor-tissue blocks from 281 of these were available, 22 patients were not resectable. There were, however, evaluable specimens from only 244 patients, which were included in the present study. Disease stage was based on the tumor, node, metastasis-stage (TNM-stage) (International Union Against Cancer [UICC] [http://www.uicc.org/resources/]). Citrate plasma samples were collected from the CRC patients before large bowel endoscopy as described previously 13.

The clinical data for these patients are presented in Table1. Use of the patient material was approved by The Regional Ethical Committee of Copenhagen and Frederiksberg (KF 01-080/03) and the Danish Data Protection Agency (2003-41-3312) approved the protocol and the study was carried out according to the Helsinki Declaration II.

**Table 1:** Patients' characteristics

		*N* (%)	Association^1^
Age	70.5 (32.7–91.7)^2^		0.46, 0.07, 0.30^3^
			0.42, 0.11, 0.48
Gender	M	148 (61)	0.10, 0.86, 0.94^4^
	F	96 (39)	0.74, 0.33, 0.66
Localization	Right-sided colon cancer	56 (23)	
	Left-sided colon cancer	95 (39)	0.039, 0.003, 0.011^4^
	Rectal cancer	93 (38)	0.12, 0.39, 0.09
Stage	I	41 (17)	
	II	82 (34)	0.20, 0.67, 0.80^4^
	III	66 (27)	0.81, 0.45, 0.51
	IV	45 (18)	
	Not staged	10 (4)	
Chemotherapy	No	183 (75)	0.008, 0.09, 0.001^4^
	Yes	61 (25)	0.005, 0.64, 0.049

1 *P*-values for the association between the clinical variable and uPAR-scores for the cancer cells/core, macrophages/core, myofibroblasts/core, cancer cells/invasive front, macrophages/invasive front and myofibroblasts/invasive front, respectively.

2 Median age and range.

3 *P*-values for Spearman rank correlations.

4 *P*-values for the Wilcoxon rank-sum test.

### Antibodies

Rabbit polyclonal antibody (pAb) and a mouse monoclonal antibody (mAb) (clones R2, IgG1) against uPAR have been described previously 15,16. MAbs against pan-cytokeratin (pan-CK) (clone AE1/AE3) and CK20 (clone K_s_20.8) for detection of cancer cells, CD68 for detection of macrophages (clone PG-MI), and *α*-smooth-muscle-actin (*α*-SMA) for detection of myofibroblasts (clone 1A4)—as well as EnVision horseradish peroxidase Mouse (K4001), EnVision horseradish peroxidase Rabbit (K4003) secondary antibodies, and an EnVision™ G│2 Double Staining Kit (K5361) were purchased from Dako (Glostrup, Denmark).

### Immunoperoxidase staining

Three-micrometer paraffin sections from each of the blocks were deparaffinized with xylene and hydrated through ethanol/water dilutions. Sections, which were stained with uPAR-antibodies (pAb and R2), were pretreated with Proteinase K (5 *μ*g/*μ*L) in a Proteinase K-buffer (50 mmol/L Tris-HCl, 50 mmol/L EDTA, pH 8.0) at 37°C for 15 min, and sections stained with the *α*-SMA antibody were pretreated at 98°C in TEG-buffer (10 mmol/L Tris-HCl, 0.5 mmol/L EGTA, pH 9.0) for 10 min using a T/T Micromed microwave processor (Milestone, Sorisol, Italy). Immunohistochemical stainings were performed using a LabVision Autostainer 360 (LabVision, Freemont, CA). The autostainer was programmed with two drop zones per section; each with 100 *μ*L. Endogenous peroxidase was blocked by incubation in 1% H_2_O_2_ for 15 min and thereafter rinsed in Tris-buffered saline (TBS-T, 50 mmol/L Tris-HCl, 150 mmol/L NaCl, 0.5% Triton X-100, pH 7.6). The primary antibodies were diluted in Antibody Diluent with Background-Reducing Components (S3022, Dako) at the following concentrations: uPAR pAb (2.8 *μ*g/mL), uPAR clone R2 (0.86 *μ*g/mL), *α*-SMA antibody (0.35 *μ*g/mL), and added to the section. After 30 min incubation the primary antibodies were detected with EnVision Rabbit or Mouse reagents for 30 min. The sections were then developed with NovaRed (Vector Laboratories, Burlingame, CA) for 15 min. Each incubation step was followed by washes in TBS-T. Finally, the sections were counterstained using ½× Mayer's hematoxylin for 1 min, and thereafter removed from the autostainer and dehydrated in ethanol solutions and mounted with pertex using a CoverSlipper from Dako.

### Double immunohistochemistry

Paraffin sections of 3 *μ*m were double stained using antibodies against CD68 and CK-mix (CK-pan + CK20). Stainings were performed with the EnVision™ G│2 Double Staining Kit using the protocol provided by the manufacturer. Antigen retrieval was performed with Proteinase K (5 *μ*g/*μ*L) in a Proteinase K-buffer at 37°C for 15 min. After pretreatment, the slides were mounted on Shandon racks with immunostaining cover plates (Thermo Shandon, Pittsburgh, PA). Subsequently, the endogenous peroxidase activity was blocked by incubation with H_2_O_2_ provided in the kit for 15 min. The antibody against CD68 (0.30 *μ*g/mL) was diluted in Antibody Diluent with Background-Reducing Components and added to the slide and incubated for 2 h at room temperature. The detection was done with a secondary antibody and then developed with 3.3-diaminobenzidine (DAB). Thereafter, the second primary antibody (CK-pan [0.54 *μ*g/*μ*L], CK20 [0.68 *μ*g/*μ*L]) was added to the slides and incubated overnight at 4°C. The second primary antibody was detected with a secondary antibody, which then was developed with Permanent Red. The sections were counterstained using 150 *μ*L Mayer's hematoxylin for 30 sec and were finally dehydrated in an oven at 60°C for 1 h before coverslips were mounted using a Dako CoverSlipper.

### Scoring

The sections stained for uPAR using the anti-uPAR pAb were coded and evaluated blindly by two experienced pathologists (O. D. L. and J. P. H.). uPAR is expressed by circulating neutrophils. These served as internal positive control for the uPAR-stainings 17. Sections with uPAR-negative neutrophils were restained.

uPAR-immunoreactivity was scored separately in cancer cells, macrophages, and myofibroblasts, as described previously 6,7. These cell types were identified in neighboring sections by immunohistochemical stainings for CKs (cancer cells) and CD68 (macrophages), and *α*-SMA (myofibroblasts). The counting of uPAR-positive cells was performed independently in two locations of the tumors, the invasion zone (defined up to 0.5 mm broad in the tumor periphery), and in the tumor core (everything else but areas of necrosis, see also 6,7). The percentages of uPAR-positive cells were grouped into the following categories: 0, no uPAR-positive cells detected; 1, less than 1% positively stained cells; 2, between 1% and 5% positive cells; 3, between 5% and 10% positive cells and 4, more than 10% positively stained cells.

In addition to the uPAR-scoring, the size of the CD68-positive macrophages was evaluated by two independent observers (O. D. L. and M. I.). Macrophages smaller than the diameter of two leukocytes were considered small, whereas macrophages exceeding the diameter of two leukocytes were considered large.

### Measurements of uPAR-forms

The levels of the different soluble uPAR-forms in all the patient citrate plasma samples were measured using time-resolved fluorescence immunoassays 13.

### Statistics

The uPAR-scores were dichotomized as negative (score = 0) and positive (score > 0). Comparisons between the uPAR-scores, the actual score as well as the dichotomized scores were compared to the clinical covariates using rank statistics. Interobserver agreement was evaluated using the Spearman rank correlation, tests for symmetry, and weighted Kappa statistics. Interobserver agreement for CD68 measurements was assessed by Kappa statistics with 95% confidence limits. The measured uPAR-forms [uPAR(I–III), uPAR(I–III)+uPAR(II–III) and uPAR(I)] were all analyzed on the log scale (base 2) (resulting hazard ratio [HR] in twofold difference in the marker levels). Survival probabilities for time to death of all causes have been estimated employing the Kaplan–Meier method. The association between the clinical covariates and the uPAR-scores has been done using the Cox proportional hazards model for univariate as well as multivariable analysis. Multivariable analysis of the uPAR-scores included only those covariates that were statistically significant in the univariate setting. Results are presented by the HR with 95% confidence interval (CI) and a generalized concordance-index (C-index) 18. The assumptions of the regression models were assessed using martingale and Schönfeld residuals and 10-fold internal cross-validation. *P*-values less than 5% were considered significant. Statistical calculations have been done using SAS (v9.2, SAS Institute, Cary, NC) and R (R Core Team [2013]. R: A language and environment for statistical computing. R statistical Computing, Vienna, Austria. URL. http://www.R-project.org/). The results of this project are reported in accordance with the REMARK guidelines 19.

## Results

### uPAR-immunohistochemistry

**Figure 1:**
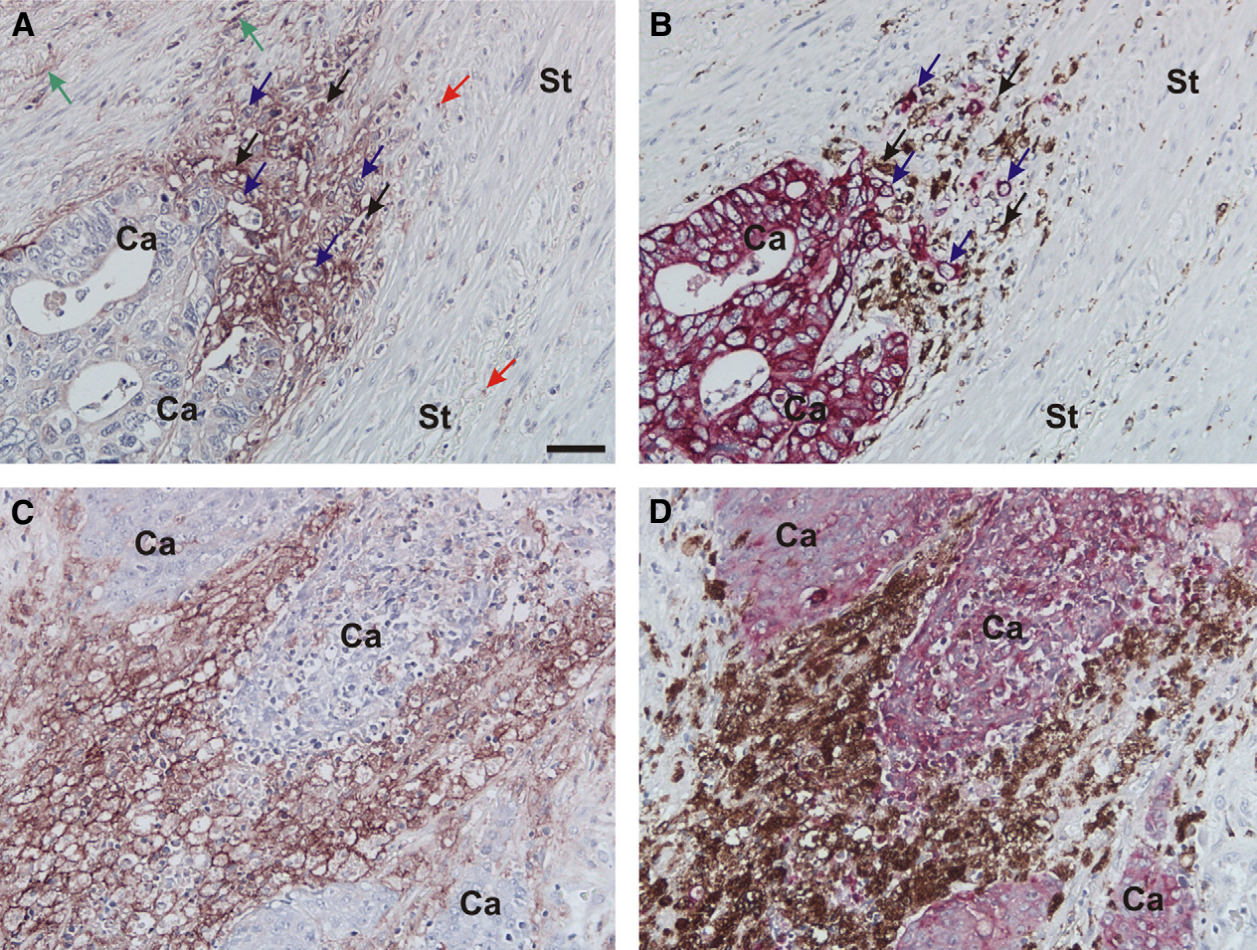
Immunohistochemical identification of macrophages, cancer cells and uPAR-expressing cells. Adjacent tissue sections of CRC were processed for either uPAR-immunohistochemistry using a pAb against uPAR (A and C), or double immunohistochemistry for CKs and CD68 using the Envision G*|*2 Double System kit from Dako (B and D). The uPAR-stainings are visualized with NovaRed, the CK-stainings with Permanent Red and the CD68-stainings with DAB. Intense uPAR-immunoreactivity is seen at the invasive front primarily in cells identified as macrophages (black arrows in A and B) but also in some budding cancer cells (blue arrows in A and B), and some myofibroblasts (green arrows in A). uPAR-immunoreactivity is also seen in neutrophils scattered throughout the tissue (red arrows in A). Strong uPAR-immunoreactivity is often seen in large macrophages within the tumor core (C and D). Bar in A: ∼50 *μ*m. DAB, diaminobenzidine; uPAR, urokinase-type plasminogen activator receptor; CRC, colorectal cancer.

Sections from each of the 244 biopsies of CRC were processed for immunohistochemistry using the pAb and mAb against uPAR. Identical stainings were seen for the two uPAR-antibodies, and sections stained with pAb against uPAR were used for further analysis. Immunohistochemical stainings for specific cell markers, such as CKs for cancer cells and CD68 for macrophages (double staining) and *α*-SMA for myofibroblasts were in addition performed on adjacent sections (Fig.1).

When evaluating the CK-stained sections, we found that no tumor invasive front was present in specimens from two of the patients. These samples were censored, therefore, the invasive front have been analyzed in 242 specimens and tumor core in all 244 evaluable specimens. The pAb against uPAR has been validated in earlier studies 6,7,9.

uPAR-immunoreactivity was seen in primarily tumor-associated macrophages (TAMs) at the invasive front in 183 of the 242 biopsies (Fig.1A and B). Macrophages positive for uPAR were also observed in the tumor core in 176 of the cases and also seen in macrophages in luminal parts of the cancer glands and in areas with necrosis (Fig.1C and D). uPAR-positivity was also seen in budding cancer cells located at the invasive front in 195 of the cases as well as in the tumor core in 150 of the cases (Fig.1A and B). Myofibroblasts located at the invasive front were uPAR-positive in 208 of the cases and myofibroblasts located at the tumor core were positive in 134 of the cases (Fig.1A and data not shown). uPAR-immunoreactivity was also observed in the endothelial cells located in vascular structures of the submucosa and in distal and proximal nerve bundles located in the submucosa. In 21 of the cases, uPAR-immunoreactivity was only seen in infiltrating neutrophils scattered throughout the tissue. The finding described above confirms previous localization studies in human CRC 8,9.

### Scoring of uPAR-positive cells

In order to validate the uPAR-scoring of the specimens by (O. D. L.) the other trained pathologist (J.P.H.) scored specimens from 71 patients for uPAR-positivity on each of the three cell types at the invasive front and in the tumor core. Statistical examination of the agreement between the different uPAR-scores is shown in Table2. These results suggest a moderate agreement between the observers, although there was a slight systematic difference. Statistical analysis of the second observer's scores demonstrated similar trends as the first observer (data not shown). The first observer's (O. D. L.) uPAR-scorings were then used for further analysis.

**Table 2:** Interobserver agreement

	Spearman rank correlation	Test for symmetry (*P*-value)	Weighted Kappa	95% CI for Kappa
Cancer cells/tumor core	0.61	0.026	0.41	0.27–0.54
Macrophages/tumor core	0.58	0.25	0.38	0.24–0.53
Myofibroblasts/tumor core	0.53	0.006	0.37	0.23–0.51
Cancer cells/invasive front	0.69	0.0007	0.51	0.38–0.64
Macrophages/invasive front	0.57	0.09	0.41	0.25–0.57
Myofibroblasts/invasive front	0.66	0.08	0.50	0.36–0.63

The Spearman rank correlations between the semiquantitative scores of uPAR assessed by two observers are shown in the first column, the second column gives *P*-values for tests of symmetry for these scores and the third measure is the weighted Kappa statistic with 95% CI.

The number of uPAR-positive cells of any of the three types was higher at the invasive front than in the tumor core, and in both locations, the scores for the macrophages were the highest. Median scores were 1 for cancer cells/tumor core, 2 for macrophages/tumor core, 1 for myofibroblasts/tumor core, 3 cancer cells/invasive front, 4 for macrophages/invasive front, and 3 for myofibroblasts/invasive front (Table2). For statistical analysis, however, the uPAR-scores were dichotomized as negative (score = 0) and positive (score > 0). The clinical covariates and *P*-values for their association with the uPAR-scores are presented in Table1. Significant associations between the uPAR-scores for tumor core are seen for cancer location with colon cancer patients having higher scores than those with rectal cancer. The rank correlations between the uPAR-scores are considered moderate (0.28–0.64).

**Figure 2:**
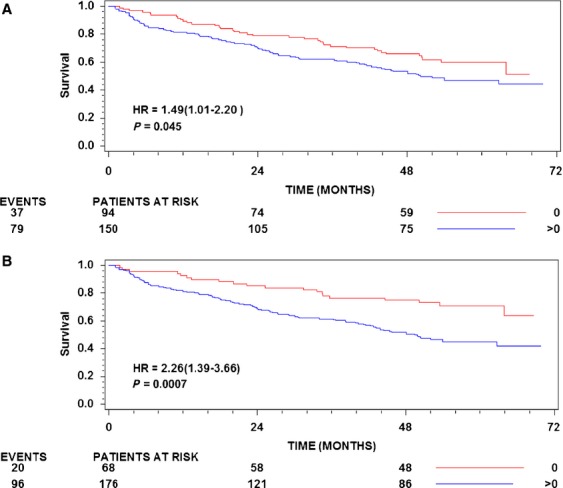
Kaplan–Meier estimates of survival probabilities. (A) uPAR on cancer cells in the tumor core stratified by uPAR-score 0 versus uPAR-score 1–4. (B) Macrophages in the tumor core stratified by uPAR-score 0 versus uPAR-score 1–4. The numbers of patients at risk at 0, 24, and 48 months are shown below the abscissa for each stratum. In addition, the number of deaths in each group is shown to the left. The *P*-values shown are for the log rank statistic. uPAR, urokinase-type plasminogen activator receptor.

In an univariate analysis, the number of uPAR-positive versus uPAR-negative macrophages (HR = 2.26 [95% CI: 1.39–3.66, *P* = 0.0009]) and cancer cells (HR = 1.49 [95% CI: 1.01–2.20, *P* = 0.047]) located in the tumor core were significantly associated to overall survival, while other parameters were not significant (Table3). Kaplan–Meier estimates of survival probabilities for these are shown in Figure2.

**Table 3:** Association of uPAR-scores to overall survival. Univariate analysis

		Positive (%)	Hazard ratio	95% CI	*P*-value
Tumor core	Cancer cells	61	1.49	1.01–2.20	0.047
	Macrophages	72	2.26	1.39–3.66	0.0009
	Myofibroblasts	55	1.27	0.88–1.84	0.21
Invasive front	Cancer cells	81	0.94	0.60–1.48	0.80
	Macrophages	72	0.77	0.52–1.15	0.21
	Myofibroblasts	86	1.54	0.85–2.81	0.16

The proportion of positive scores for each cell type for the tumor core and invasive front are shown with the results of the univariate analysis of overall survival presented by the hazard ratio with 95% CI comparing positive to negative scores and the *P*-value. uPAR, urokinase-type plasminogen activator receptor.

In a multivariate analysis, uPAR-positive versus uPAR-negative macrophages located in the tumor core showed the best separation of patients with good and bad prognosis (HR = 1.84 [95% CI: 1.12–3.04, *P* = 0.017]). There was no association or interaction between number of uPAR-positive cells and stage, cancer location, gender, and age (Table4).

**Table 4:** Multivariable analysis

Covariates		uPAR-scoring included			uPAR-scoring and plasma uPAR-forms included		
		Hazard ratio	95% CI	*P*-value	Hazard ratio	95% CI	*P*-value
Age pr. 10 years		1.29	1.08–1.54	0.005	1.17	0.98–1.40	0.09
Gender	M	1.51	1.00–2.27	0.048	1.65	1.10–2.47	0.015
	F	1	1		1	1	
Localization	Right colon cancer	2.13	1.24–3.68	0.020	1.55	0.86–2.78	0.32
	Left colon cancer	1.29	0.80–2.08		1.15	0.70–1.90	
	Rectal cancer	1	1		1	1	
Stage	I	1	1	<0.0001	1	1	<0.0001
	II	1.17	0.56–2.44		1.04	0.50–2.17	
	III	2.71	1.30–5.66		3.12	1.48–6.58	
	IV	10.28	4.68–23		8.59	3.88–19	
Chemotherapy	No	1	1	0.067	1	1	0.27
	Yes	0.61	0.36–1.03		0.74	0.43–1.27	
Cancer cells tumor core	Neg			0.26^1^			
	Pos						
Macrophages tumor core	Neg	1	1	0.017	1	1	0.023
	Pos	1.84	1.12–3.04		1.81	1.08–3.01	
uPAR(I–III) + uPAR(II–III)	Log_2_						0.14^2^
uPAR(I)	Log_2_				2.68	1.90–3.79	<0.0001

1 *P*-value to include in the model. Excluding uPAR-positive macrophages in the tumor core from the analysis results in uPAR-positive cancer cells in the tumor core being included with HR = 1.55 (95% CI: 1.03–2.34), *P* = 0.036.

2 *P*-value to include in the model. Excluding uPAR(I) from the model results in uPAR(I–III)+uPAR(II–III) being included, HR = 2.46 (95% CI: 1.81 to 3.35), *P* < 0.0001.

### Scoring of CD68-positive macrophages

Because the highest uPAR-scores were obtained for macrophages in both tumor core and at the invasive front, we scored macrophages (CD68-positive cells) (Fig.1B and D) according to size on specimens from 201 patients. The results were assessed by two observers (O. D. L. and M. I.). In the tumor core, observer 1 (O. D. L.) found 35% and observer 2 (MI) found 31% of the patients with large macrophages. At the invasive front, observer 1 found large macrophages in 75% of the patients and observer 2 in 68%. There was no association between the ratio of large to small macrophages and survival [for the tumor core HR = 1.38 (95% CI: 0.94–2.03, *P* = 0.10) and HR = 0.84 (95% CI: 0.52–1.35, *P* = 0.47) for the invasive front]. The results showed, however, that a higher proportion of large macrophages were uPAR-positive (data not shown). The observation of large macrophages in some of the biopsies may reflect a higher phagocytotic activity, which may therefore be so-called M2-polarized macrophages 20.

### Soluble uPAR measurements

The rank correlation between uPAR(I) and uPAR-immunohistochemistry range from −0.03 to 0.10, uPAR(III) and uPAR-immunohistochemistry range from 0.06 to 0.12, and uPAR(I–III) + uPAR(II–III) range from 0.06 to 0.16. The prognostic significance of the intact and cleaved soluble uPAR-forms has previously been determined in citrate plasma from 298 patients, including the 244 patients in the present study 13. Including uPAR(I–III) + uPAR(II–III) in the multivariate model showed that the soluble form was significantly (HR = 2.46 95% [CI: 1.81 to 3.45, *P* < 0.0001]) and uPAR-positive macrophages/tumor core remained significantly related to overall survival (*P* < 0.0001). Similarly for uPAR(I), the results were HR = 2.68 (95% CI: 1.90–3.79, *P* < 0.0001) and uPAR-positive macrophages/tumor core remained significant (*P* = 0.023). These results demonstrate that the uPAR-positive macrophages in tumor-tissue and the soluble uPAR-forms in plasma are independent variables. The uPAR-expressing macrophages in tumor core and uPAR(I) are additive in the model with a nonsignificant interaction term (*P* = 0.90) and thus there is no evidence of a synergistic effect. No interaction between uPAR-expressing macrophages/tumor core and stage was demonstrated (*P* = 0.76) suggesting that this is additive to that of stage.

**Figure 3:**
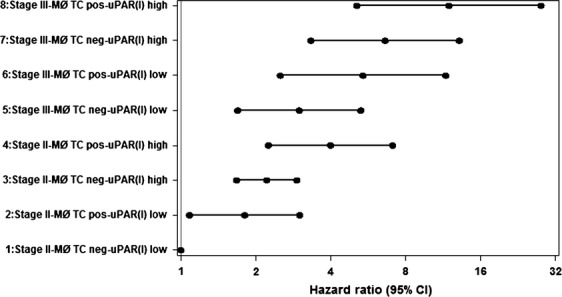
Forest plot showing estimated HRs with 95% CI for stage II or III, ± uPAR-positive macrophages in tumor core and level of uPAR(I) in plasma at the first (23.9 pmol/L) and third quartiles (41.8 pmol/L). uPAR, urokinase-type plasminogen activator receptor; HR, hazard ratio; MØ, macrophages; TC, tumor core.

Using the multivariate analysis to stratify the patients according to stage, uPAR-positive macrophages in tumor core and level of uPAR(I) in plasma resulted in a remarkable separation, the HRs for a patient with uPAR-positive/negative and uPAR(I) equal to the first and third quartile for stages II and III are shown (Fig.3). A patient with stage III and uPAR-positive macrophages/tumor core and high uPAR(I) level had a 12-fold (95% CI: 9.3–15.4) higher hazard than a patient in stage II with negative macrophage/tumor core score and a low uPAR(I) level. The C-indices for the multivariate model were 0.76 in stage I, 0.79 in stage II, 0.77 in stage III, and 0.80 in stage IV suggesting that the model predicts patient outcome with reasonable accuracy. There was no significant interaction for overall survival between adjuvant chemotherapy and uPAR-positive/negative and uPAR(I) (*P* = 0.23 and *P* = 0.73, respectively), showing the association of the two uPAR-markers to overall survival was independent of adjuvant chemotherapy (stage I–III).

## Discussion

In the present work, we show that a high uPAR-score on macrophages in the tumor core is an indicator of poor prognosis. Although significant, uPAR-positive cancer cells had less prognostic impact (Fig.2A and B). These circulating uPAR-forms are also strong prognostic markers and independent of the glycolipid-anchored uPAR on macrophages in tumor core (Table4).

There are several factors that can explain why different molecular forms of uPAR are independent prognostic markers. For the cell surface-bound uPAR-forms, we have restricted the analysis of association with prognosis to specific cell types and specific locations in the heterogeneous primary tumor. In contrast, blood is homogeneous and contains soluble uPAR-forms most likely derived from all uPAR-expressing cells in the body by at least two different mechanisms. uPAR(I) is released into the blood by proteolytic cleavage of intact uPAR on the cell surface. The two glycolipid-anchored uPAR-forms can be shed from the cell surface, thereby entering the blood 11.

In CRC uPAR is mainly expressed by macrophages at the invasive front 9. We found, however, only a significant correlation with prognosis of uPAR-expressing macrophages in the tumor core. As the invasion zone is rather narrow, in our definition less than 0.5 mm, while tumor core represents the bulk of a large, solid tumor, lack of significance for the invasion zone is not surprising.

This supports that the so-called TAMs can exert important functions in the microenvironment of CRC. Our hypothesis is therefore that polarization of an abundance of so-called M1-macrophages peripherally may lead to antitumoral capability, while the bulk of the intratumoral macrophages become tumor-promoting M2-macrophages. These processes are known to be governed by cytokines. TAMs may therefore have fundamental modulating effects on the neoplastic cell population, including tumor cell growth, cell migration, and invasion as well as angiogenesis 20,21. It follows that a high density of such macrophages is associated with a poor prognosis in most malignant tumors 22.

The pattern of uPAR-expression on the cell surface in colorectal carcinomas is similar to the expression and correlation with poor survival in esophageal and gastric adenocarcinomas 6,7. There are, however, important differences. In the esophageal cancers, poor prognosis is related to a high uPAR-score on cancer cells in the tumor core and a high percentage of uPAR-positive macrophages at the narrow invasion zone in the periphery of the tumor. In contrast, in gastric cancer the prognosis is worst, when a high number of cancer cells at the invasion zone are uPAR-positive. At the same time, overall survival in gastric and particularly in esophageal adenocarcinomas is considerably poorer than in CRC. Expression of uPAR on myofibroblasts was not associated to survival in any of these three cancer types of the GI-tract (Table3) 6,7. Thus, an explanation for this survival difference may be that in CRC, the invasion zone is dominated by tumor-inhibitory macrophages 23. Therefore, elucidation of such biological differences between malignant neoplasms of the same histological type in various locations of the same organ system—here the GI-tract—may be of clinical value.

It is worth noting that the association of uPAR-expressing cancer cells and macrophages to survival was independent of stage. This indicates that plasminogen activation with all the successive effects is an inherent property of the tumor from the beginning of malignant growth until the end stage. It is already shown that in esophageal carcinomas, premalignant lesions were uPAR-negative until early stromal invasion occurred 7. Therefore, high expression of this receptor may be important for early cancer dissemination.

Recently, a large study on the presence of disseminated tumor cells in the bone marrow at operation for CRC has been reported 24. Survival was found to be gradually lower over an observation time of 6 years, when carcinoma cells had been retrieved in the bone marrow. This indicates that an important part of the tumor dissemination occurs at an early clinical stage. This could be partly explained by tumor kinase activity and early angiogenic signaling 25. It could therefore be speculated that plasminogen activation promoting early infiltration, including vascular invasion, may be one of the key factors enabling micrometastasis and further angiogenic signaling.

We conclude that in CRC a high uPAR-score on tumor-associated macrophages and to a lesser extent on the cancer cells in the tumor core is associated with poor survival. Unlike adenocarcinomas in the upper GI-tract, that is, esophagus and stomach, this outcome is not related to the peripheral invasion zone. By combining three independent variables from our multivariate model, the outcome of the CRC could be discriminated with a factor of more than 10 (Fig.3). Thus, the combination of stage, macrophage uPAR in tumor core and preoperative plasma uPAR(I) may be a promising predictor of overall survival after resection of the primary tumor.

## Appendix

The following persons participated in the Danish Study Group on Early Detection of Colorectal Cancer:

Lars Nannestad Jørgensen, Department of Surgical Gastroenterology, Bispebjerg Hospital, Copenhagen; Jesper Olsen, Department of Surgical Gastroenterology, Glostrup Hospital, Glostrup; Hans B. Rahr, Department of Surgical Gastroenterology, Odense Hospital, Odense; Knud T. Nielsen, Department of Surgery, Randers Hospital, Randers; Søren Laurberg, Department of Surgical Gastroenterology, Aarhus Hospital THG, Aarhus and Nils Brünner, Institute of Veterinary Pathobiology, University of Copenhagen, Frederiksberg, Denmark.
